# What are the most frequent diagnoses in adolescence? The reality of an Adolescent Medicine Clinic

**DOI:** 10.1590/S1679-45082018AO4225

**Published:** 2018-06-15

**Authors:** Nina Abreu, Inês Dias, Mafalda Cascais, Alexandra Luz, Pascoal Moleiro

**Affiliations:** 1 Centro Hospitalar Leiria, Leiria, Portugal.

**Keywords:** Adolescent health, International Classification of Diseases, Referral and consultation, Saúde do adolescente, Classificação Internacional de Doenças, Encaminhamento e consulta

## Abstract

**Objective:**

To characterize the care flow and the primary diagnoses of an Adolescent Medicine Clinic.

**Methods:**

A retrospective descriptive study, with analysis of clinical processes of adolescents (10-18 years) seen at the Adolescent Medicine Clinic, from January 2006 to December 2013. The following variables were analyzed: sex, age, number of visits, referring service and primary diagnoses according to the International Statistical Classification of Diseases and Related Health Problems. As to the variable age, the adolescents were divided into two groups: Group I comprised those aged 10-14 years, and Group II, 15-18 years.

**Results:**

A total of 7,692 visits were carried out, in that, 1,659 first visits (22%), with an annual growth rate of 6%. The mean age was 14.2 years, and 55% of patients were female. The group of endocrine, nutritional and metabolic diseases was the most representative in our sample (34%), with obesity being the most frequent diagnosis in both sexes and age groups (23%), with a higher prevalence in males (13% male *versus* 10% female, p<0.001) and younger adolescents (18% in Group I *versus* 5% in Group II p<0.001). The group of mental and behavioral disorders was the second most prevalent (32%), affecting mainly females (39% female *versus* 22% male, p<0.001) and the older age group (39% Group II *versus* 27% Group I, p<0.001). Social problems were the primary diagnosis in 8% of visits.

**Conclusion:**

Most diseases diagnosed have a strong behavioral and social component, particularly mental disorders and obesity. This specific type of diagnoses reinforces the need for a global approach for adolescents and specialized adolescent medicine units/clinics.

## RESUMO

**Objetivo:**

Caracterizar o movimento assistencial e conhecer os diagnósticos principais de uma consulta de Medicina do Adolescente.

**Métodos:**

Estudo descritivo retrospectivo, com análise dos processos clínicos dos adolescentes (10 a 18 anos) seguidos na consulta de Medicina do Adolescente, de janeiro de 2006 a dezembro de 2013. Foram analisadas as variáveis: sexo, idade, número de consultas, entidade que referenciou e diagnósticos principais, de acordo com a Classificação Estatística Internacional de Doenças e Problemas Relacionados com a Saúde. Com relação à variável idade, os adolescentes foram divididos em dois grupos: Grupo I, que incluiu adolescentes de 10 a 14 anos, e Grupo II, que incluiu aqueles com 15 aos 18 anos.

**Resultados:**

Realizaram-se 7.692 consultas, sendo 1.659 (22%) primeiras consultas, com taxa de crescimento anual de 6%. A média de idade foi de 14,2 anos, com 55% do sexo feminino. O grupo das doenças endócrinas, nutricionais e metabólicas foi o mais representativo da amostra (34%), sendo a obesidade o diagnóstico mais frequente em ambos os sexos e grupos etários (23%), com maior prevalência nos rapazes (13% homens *versus* 10% mulheres; p<0,001) e nos adolescentes mais jovens (18% do Grupo I *versus* 5% do Grupo II; p<0,001). O grupo dos transtornos mentais e do comportamento foi o segundo mais prevalente (32%), afetando principalmente as adolescentes (39% mulheres *versus* 22% homens; p<0,001) e o grupo etário mais velho (39% do Grupo II *versus* 27% do Grupo I; p<0,001). Problemas sociais constituíram o principal diagnóstico em 8% das consultas.

**Conclusão:**

A maioria das patologias diagnosticadas tem forte componente comportamental e social, com destaque para as doenças mentais e a obesidade. Esta tipologia específica de diagnósticos evidencia a necessidade de uma abordagem global do adolescente e de unidades/consultas especializadas nesta faixa etária.

## INTRODUCTION

Adolescence is a period in which it is possible to promote health, as there are several physical, psychological and social changes, as well as new life habits being developed with short- and long-term implications, which determine the future health standards of adults.^(^ ^1^ ^–^ ^3^ ^)^

Evidence suggests that an early and adequate intervention in adolescence provides long-term health and socioeconomic benefits.^(^ ^4^ ^–^ ^6^ ^)^ The World Health Organization (WHO) increasingly reinforces the importance of “adolescent-friendly” health services.^(^ ^4^ ^)^ Working specifically in the adolescent age group is critical from the public health perspective for improving the global health landscape, particularly in some fields, such as mental health, accidents and chronic diseases,^(^ ^3^ ^)^ which are gaining increasing relevance.

Despite the evidence described, the adaptation of health systems to support this view has been scarce and insufficient.^(^ ^7^ ^)^ In most countries, the adolescent population still finds, in hospitals and primary care facilities, an environment that does not cater to their specific characteristics and needs.^(^ ^5^ ^)^ This lack of individualized care drives adolescents away from health services, and wastes opportunities to promote health and prevent risky behaviors.

One reason for this insufficient response may be the scarcity of general data on what is diagnosed at the level of health services. Despite the studies addressing specific issues of adolescence, few present an overview of adolescent disease profiles, Adolescent Medicine appointments and the most commonly diagnosed diseases. These data are fundamental for health services to adjust to the needs of adolescents.

Also, there are substantial differences in the definitions and methods used in each study. The use of a universal and validated classification for the diseases identified was infrequent, which makes it impossible to compare between countries and over time.^(^ ^7^ ^)^ Strategies to standardize coding practices in different countries must be a priority,^(^ ^2^ ^)^ since validated information is critical for pursuing more rigorous health programs and policies that cater to the needs of adolescents.^(^ ^4^ ^.^ ^7^ ^)^

The International Statistical Classification of Diseases and Related Health Problems (ICD) is a standard diagnostic tool implemented by the WHO to standardize the data collected by different health entities, allowing for consistent comparison, analysis and compilation of said data.^(^ ^8^ ^,^ ^9^ ^)^ It is a consensual and internationally accepted language, which can help improve adolescent care.

This study aimed to elucidate the disease profile of adolescents using hospital services and standardize the coding of health data - to ultimately help improve the care provided to this age group.

## OBJECTIVE

To characterize the care processes of the clinic and define the primary diagnoses according to the International Statistical Classification of Diseases and Related Health Problems.

## METHODS

A retrospective, descriptive and cross-sectional study, with analysis of clinical processes of adolescents (aged 10 to 18 years) seen at the Adolescent Medicine Clinic (AMC) of a general hospital, in Portugal, from January 2006 to December 2013. The AMC was implemented in this hospital in August 2005, with two designated full-time physicians and one nurse, and was supported by other specialties. The clinic provides care to adolescents with medical disorders, referred by other units of the pediatrics service, other hospital services and out-of-hospital units.

The following variables were analyzed: age, sex, number of visits (total/year, first and follow-up), referring entities (hospital, primary care center, others) and primary diagnoses. Two age groups were created: ages 10 to 14 years (Group I) and 15 to 18 years (Group II).

The diagnoses were coded according to version 10 of the ICD (ICD10), and were later compiled into large groups, using the same classification. Obesity was defined as body mass index (BMI) >95th percentile, based on the Center for Disease Control and Prevention (CDC) age-and sex-specific growth charts of year 2000.

Adolescents with no recorded diagnoses or who had not yet been diagnosed were excluded.

Statistical analysis of the data was performed on IBM^®^ Statistical Package for Social Sciences^®^ (SPSS^®^), version 22.0. In the comparative analysis, we used the χ^2^ test. Statistical significance was defined as p value <0.05.

The study was approved by the Institutional Review Board of *Centro Hospitalar de Leiria*, Portugal. Confidentiality, anonymous data and professional secrecy were ben preserved and there were no ethical issues involved.

## RESULTS

During the study period, there were 7,692 visits (9% of total pediatric visits of the hospital for the same period). Of these, 1,659 (21.6%) were first visits and 6,033 (78.4%) were return visits, corresponding to an average care flow of 962 visits/year. The average annual growth rate was 6%, with an increase of about 54% in the total number of visits between 2006 and 2013, the year with the highest number of visits (n=1,169). The proportion of first visits varied between 16 and 26% of the total, with an average of 207 visits/year and an average annual growth of 9%.

Twenty-one percent of adolescents were referred by primary care centers (referral average of 43 adolescents per year), and the referral ratio from the primary care center/hospital was 1:4. Most referrals were female adolescents (58% *versus* 42%, p=0.01) and in the age group of 10 to 14 years (58% *versus* 42%, p=0.01). Please note that the sex-based difference in referrals can also be seen in the younger age group (55% for girls *versus* 45% for boys, p=0.01), although it is more marked in the older age group (62% for girls *versus* 38% of boys, p=0.01).

Regarding the characteristics of the population followed-up over these 8 years, the mean age was 14.2 years, 55% (n=4,239) of adolescents were female and 51% (n=3,925) fit into Group I.

During the study period, a total of 1,136 primary diagnoses (68% of total first visits), corresponding to 1,118 adolescents. The total number of diagnoses was higher than the number of patients because 18 adolescents had more than one first visit (after discharge, they were referred back for a different reason). On the day of data collection, 523 adolescents were undergoing diagnostic investigation and/or still undiagnosed.

The group of endocrime, nutritional and metabolic diseases (ENMD) was the most representative in the total sample (34%). This group of diseases was also the most frequent in males (40%) and in Group I (44%). Among females and in Group II, mental and behavioral disorders (MBD) were the most prevalent (39% in girls and 39% in Group II). This age and sex-based distribution trend was statistically significant in both disease groups, as shown in table 1.

**Table 1:** Groups of most frequent diagnoses, by age and sex

Groups (ICD10)	n (%)	Age				Sex		
		χ (δ)*	Group I† (%)	Group II‡ (%)	p value	Male n (%)	Female (%)	p value
Endocrine, nutritional and metabolic diseases (IV)	390 (34)	13 (±2)	44	22	<0.001	40	31	0.002
Mental and behavioral disorders (V)	361 (32)	15 (±2)	27	39	<0.001	22	39	<0.001
Factors that influence health status and contact with health services (XXI)	119 (10.5)	14 (±2)	10	12	0.220	12	10	0.344
Diseases of the genitourinary system (XIV)	66 (5.8)	13 (±2)	7	4	0.090	7	5	0.066
Diseases of the respiratory system (X)	47 (4)	14 (±2)	4	4	0.763	5	4	0.367
Diseases of the digestive system (XI)	42 (3.7)	14 (±2)	4	4	0.637	5	3	0.203
Diseases of the nervous system (VI)	33 (2.9)	15 (±2)	2	6	0.004	2	3	0.372
Diseases of the blood and blood-forming organs and certain disorders involving the immune mechanism (III)	20 (1.8)	15 (±2)	1	3	0.112	2	2	0.822
Diseases of the musculoskeletal system and connective tissue (XIII)	14 (1.2)	15 (±2)	1	2	0.591	2	1	0.593
Diseases of the skin and subcutaneous tissue (XII)	12 (1.1)	15 (±2)	1	2	0.138	1	1	0.770

Comparative analysis with χ^2^ test.

* mean age in years (standard deviation);

† 10 to 14 years;

‡ 15 to 18 years.

The group of factors that influence health status and contact with health services, *i.e*. adolescents who sought health services for some specific purpose or problem, but did not have any disease, was the third most representative (10.5%).

Table 2 shows the most frequent diagnoses. Obesity was the most prevalent for the total sample (23%) and also for both sexes (30% male *versus* 17% female) and age groups (31% Group I *versus* 12% Group II).

**Table 2:** Most frequent diagnoses, by age and sex

Diagnosis (ICD10)	n (%)	Age			Sex		
		Group I* (%)	Group II† (%)	p value	Male (%)	Female (%)	p value
Obesity (E66.0)	261 (23)	31	12	<0.001	31	18	<0.001
Eating disorders (F50)	109 (10)	8	12	0.011	4	14	<0.001
Other anxiety disorders (F41)	90 (8)	6	10	0.011	4	11	<0.001
Persons with potential health hazards related to socioeconomic and psychosocial circumstances (Z55-Z65)	90 (8)	8	8	0.761	8	8	0.833
Disorders of thyroid gland (E00-E07)	67 (6)	6	5	0.441	2	9	<0.001
Mood [affective] disorders (F30-39)	49 (4)	3	6	0.012	4	5	0.463
Somatoform disorders (F45)	42 (4)	3	4	0.339	2	5	0.006
Asthma (J45)	36 (3)	3	3	0.735	4	3	0.608
Encounter for medical observation for suspected diseases and conditions ruled out (Z03)	29 (2.5)	2	4	0.051	4	2	0.053
Migraine/other headache syndromes (G43-44)	25 (2.2)	1	3	0.038	2	3	0.413
Short stature (E34.3)	24 (2)	3	1	0.012	4	1	0.006
Excessive, frequent and irregular menstruation (N92)	17 (1.5)	1	2	0.805	-	-	-
Conduct disorders (F91)	16 (1)	2	1	0.207	2	1	0.040
Disorders of breast (N60-64)	16 (1)	2	1	0.207	3	1	0.002
Type 1 *diabetes mellitus* (E10)	12 (1)	1	1	0.770	2	1	0.138

Comparative analysis with χ^2^ test.

* 10 to 14 years;

† 15 to 18 years.

The second most frequent diagnosis for females was eating disorders (ED), in 14%; among males, problems related to socioeconomic and psychosocial circumstances (8%).

Psychosocial diseases, including all MBD (32%) and problems related to socioeconomic and psychosocial circumstances (8%) accounted for 40% of total diagnoses.

A comparison between sexes showed a statistically significant higher prevalence of ED, anxiety disorders, thyroid disease and somatic symptom disorders in girls, and obesity, short stature, conduct disorders and breast disease in boys.

When this analysis was applied to age groups, there was statistical significance for the higher prevalence of ED, anxiety disorders, mood disorders and headache in the older age group, and obesity and short stature in the younger group.

**Figure 1:**
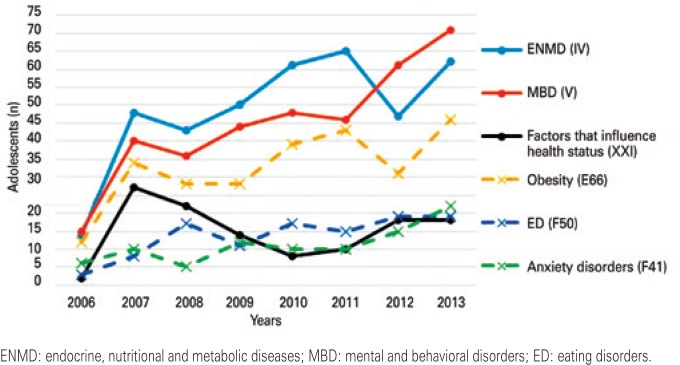
Frequency of the main groups of diagnoses and specific diagnoses

Regarding the evolution over time of the primary diagnoses, as shown in figure 1, there was a trend towards an increase in the number of diagnoses given. The mean annual growth rate of ENMD was 20% (with the obesity diagnosis growing, on average, 21% per year) and MBD was 18% (with ED growing, on average, 26% per year, and anxiety disorders, 18%).

## DISCUSSION

Adolescent Medicine is an area that has taken on an increasingly important role. In the study hospital, there was an increase of approximately 54% in total number of visits between 2006 and 2013, with a proportional increase in the number of first visits, in line with the trend in the Pediatrics service.^(^ ^10^ ^–^ ^12^ ^)^

These results reflect the progressively greater need to attend to the health needs of this age group. However, only one-fifth of the referrals were from primary care centers, with hospital services accounting for most referrals. This result may reflect (1) an insufficient response from healthcare services in the community, which are still poorly prepared and do not cater to adolescents and their specific problems; and (2) a dependency of emergency/hospital services on interventions at crisis level and routine care.^(^ ^13^ ^)^

In our sample, there was a difference in patterns of use, according to sex and age. There was a slight predominance of visits among females and in the younger age group, in agreement with the literature.^(^ ^2^ ^,^ ^14^ ^–^ ^16^ ^)^ The sex-based difference in visits was higher in Group II, and the older boys had the lowest percentage of referrals for appointment. These differences are possibly related to psychosocial aspects (sex and age group differences in beliefs, perception of illness/discomfort, approach to problems and demand for health services), and not to a lower incidence of health problems in males and in the older age group.^(^ ^13^ ^,^ ^15^ ^,^ ^16^ ^)^ In fact, in developed countries, during adolescence, the morbidity and mortality rate is higher in males, who have more risky behaviors, such as alcohol and drug consumption, accidents, violence and high-risk sexual behavior.^(^ ^2^ ^,^ ^15^ ^,^ ^16^ ^)^

Sex and age-based differences in our sample were also found at the level of diagnoses.

In boys and in Group I, ENMDs were more frequent, particularly obesity, which was the most prevalent disease in this study for both sexes and age groups. The prevalence (23%) reflects the increasing weight that this disease has in society (in Portugal, it is estimated that about 7% of adolescents are obese and in the United States, this figure rises to 21%)^(^ ^17^ ^,^ ^18^ ^)^ and the increasing trend will continue.^(^ ^18^ ^–^ ^20^ ^)^

Regarding the sex and age distribution of obesity, results were equivalent to those described in Europe, according to which the prevalence is higher in male and younger adolescents.^(^ ^17^ ^,^ ^20^ ^,^ ^21^ ^)^ The higher frequency in the younger age group, although a red flag for the increasingly earlier onset of obesity, may also encourage earlier screening/diagnosis, allowing for prompter and more effective interventions.

In females and in Group II, MBD were the most frequent disease groups - with the exception of conduct disorders, which were more frequent in males. This distribution is in line with most studies about mental disorders in adolescents.^(^ ^1^ ^,^ ^5^ ^,^ ^22^ ^–^ ^24^ ^)^

Overall, mental disorders accounted for about one-third of diagnoses (32%), with the second highest prevalence; however, if we exclude obesity from the ENMD group, MBD becomes the primary diagnosis for both sexes and age groups. In fact, in our sample, there was an up trend for mental problems over the years, and these disorders took on an increasingly relevant role in medical visits. Globally, MBD also have a central role in adolescent health, affecting 10 to 20% of adolescents on average − an American study reported a prevalence as high as 49.5%.^(^ ^5^ ^,^ ^22^ ^,^ ^24^ ^–^ ^26^ ^)^ These diseases are the main cause of disability in children and adolescents all over the world.^(^ ^27^ ^)^ The burden increases during childhood and peaks at the end of adolescence and start of adult life,^(^ ^25^ ^)^ and half of the cases identified in adults started when the individuals were 14-year old.^(^ ^1^ ^,^ ^5^ ^,^ ^28^ ^)^ Therefore, adolescence is a critical time to detect and treat mental disorders. In respect to prevention strategies, it seems important that they be put in place at an earlier stage of adolescence.

In our sample, the most common mental disorders were eating disorders (10%), followed by anxiety disorders (8%) and mood disorders (4%). In large international studies, we found similar prevalences of anxiety disorders (7 to 13%) and mood disorders (4 to 7%), but eating disorders were found in as little as 2 to 3% of adolescents.^(^ ^5^ ^,^ ^23^ ^,^ ^24^ ^,^ ^26^ ^)^ These differences may be related to the specificities of the sample, since most of the studies reviewed were carried out in the community, and our sample was taken from a population of patients attending medical consultations at a hospital. There may have been a bias in the selection of more specific diseases, such as eating disorders, which are followed-up only in specialized centers. Conduct and behavioral disorders were not so representative in our sample, and this may be due to the fact that the service investigated offers specialized consultations for attention *deficit* hyperactivity disorder (ADHD), to which most of theses cases are reffered.

The third most frequent diagnosis group in our sample included adolescents with no diseases. Within this group, social problems stood out, attesting to the weight of psychosocial disorders in adolescent health (in total, MBD and social problems corresponded to 40% of diagnoses found). This type of diagnoses points to the need for a global approach for adolescents as well as specialized health units/consultations for this age group. It is important to invest in the training of professionals that care for adolescents, particularly at community facilities. Without proper psychosocial assessment and high-quality interventions, many health problems in adolescence will be missed by practitioners and carried over to adulthood.

It's also important not to restrict adolescent interventions to the healthcare sphere. Other systems, such as education, family and social care, are important. Only with everyone's support can we move from a model of intervention-at crisis-level to one of anticipatory guidance, screening and primary prevention.

Concerning the type of intervention that should be done, our study results show that in the older age group and for females, interventions should be primarily targeted at mental health. In younger and male adolescents, interventions should focus on combating obesity/overweight.

The interpretation of data presented here must take into account some limitations of this study. The number of diagnoses recorded was limited to one (primary) for each adolescent, which may lower the true incidence of some diseases that could present as a secondary diagnosis. In order to avoid too much dispersion of the results, we decided to use larger diagnostic groups, which limited the gathering of more specific information for certain disease groups.

Another limitation was the fact that we did not include in the sample all the adolescents seen in the Pediatrics Service because there are other clinics (Pulmonology, Gastroenterology and Nephrology) that manage adolescents with diseases under these specific specialties and, therefore, some diseases would be underdiagnosed. At the moment, the AMC does not have the capacity to care for all adolescents referred to the service and must therefore, prioritize patients with disorders associated with adolescence.

It would also have been important, for better interpretation of the results, to assess the factors that determine the use of the hospital clinic.

The limitations found can be overcome by carrying out more extensive studies, looking at more variables and all hospital services/clinics that manage adolescents. It would be important for all studies to use the same internationally validated classification, as we did in this study, to facilitate comparisons. Knowing the health/disease profile of adolescents and being able to compare data by using international consensual codes enables us to better understand the reality of adolescents and take measures in consonance with their needs, which will also have positive impacts on global health in the future.

## CONCLUSION

This study documented a high prevalence of mental disorders and obesity in adolescence, tending to increase over time.

Most diseases diagnosed were behavioral and social - more than biomedicals.
